# When assessment alignment masks generalizability: a methodological cautionary tale from an OBE pilot in nephrology

**DOI:** 10.3389/fmed.2026.1893135

**Published:** 2026-07-28

**Authors:** Qi-shun Wu, Lin Wang, Gui-rui Sang

**Affiliations:** 1 Second Affiliated Hospital of Wannan Medical University, Wuhu, Anhui, China; 2 Affiliated Fourth Hospital of Jiangsu University, Zhenjiang, Jiangsu, China

**Keywords:** assessment alignment bias, counterbalanced design, far-transfer assessment, medical education research, near-transfer assessment, outcome-based education, research competency, validity threat

## Abstract

**Background:**

Research competency is increasingly recognized as essential for medical graduates, yet rigorous evaluation of educational interventions remains uncommon. Outcome-based education (OBE) aligns teaching activities with predefined competencies, but its application to research competency development in specialized clinical rotations is underexplored.

**Methods:**

This methodological reflection draws on a feasibility pilot evaluating an OBE research-integrated teaching model in nephrology. A mixed-methods quasi-experimental design was employed, combining quantitative outcome data with qualitative feedback within a within-instructor counterbalanced framework. Eight instructors taught 126 fifth-year medical students across two consecutive 4-week blocks, with model crossover and no washout period. The primary outcome was a comprehensive examination (100-point scale); secondary outcomes included self-rated research competency, capstone proposal ratings, and platform usability.

**Results:**

Near-transfer assessment scores were higher in the OBE condition (mean difference 5.6 points, 95% CI 3.1–8.1, *P* = 0.001). Sensitivity analysis excluding the research design subscale (which directly overlapped with OBE micro-projects) attenuated the effect by 32% (mean difference 3.8 points, 95% BCa CI 1.2–6.4, *P* = 0.005). Self-rated competency gains and capstone scores favored the OBE group, but all assessments lacked far-transfer tasks.

**Discussion:**

The statistically significant differences reflect near-transfer performance on examination tasks structurally aligned with training activities, not generalizable competency acquisition. The design failed to deliver credible evidence due to four fatal flaws: (1) assessment-task alignment between the research design subscale and OBE micro-projects; (2) absence of graduated transfer assessment; (3) insufficient instructor sample (*n* = 8) to detect instructor-by-model interactions; and (4) lack of a washout period introducing cross-contamination. The control condition (passive didactic teaching) conflated OBE-specific effects with generic active learning benefits. This article provides no evidence that the OBE model improves research competency; its sole contribution is to identify and warn against assessment alignment bias as a common validity threat. We propose a graduated transfer assessment framework and methodological checklist for future studies.

## 1 Introduction

Research competency is increasingly recognized as essential for medical graduates. The American Society of Nephrology has emphasized competency-based education across fellowship training levels, with research pathways identified as critical for the future workforce (1, 2). Outcome-based education (OBE) aligns teaching activities with predefined competencies (3), yet its application to research competency development in specialized clinical rotations remains underexplored in Asian contexts.

A persistent limitation of previous studies is the inability to rule out instructor effects as an alternative explanation for observed differences (4). We developed an OBE-guided research-integrated teaching model for nephrology and initially evaluated it using a within-instructor counterbalanced design, in which each instructor taught both the experimental and control curricula. This design reduces, but does not eliminate, instructor confounding.

The primary contribution of this manuscript is methodological rather than demonstrating the effectiveness of the OBE model. We explicitly position this pilot as a hypothesis-generating feasibility study with a dual purpose. First, it evaluates whether an OBE research-integrated model can be delivered within existing nephrology rotations. Second, and more importantly, it serves as a methodological cautionary tale. The design incorporates several features that preclude causal inference: non-randomized allocation, absence of a washout period, structural overlap between assessment content and intervention activities, and a small instructor sample. No causal inferences are intended. Rather, we use this study to illustrate how assessment-task alignment can render even statistically significant findings difficult to interpret as evidence of competency improvement.

This article does not demonstrate that the OBE model improves research competency. The statistically significant differences reported below reflect performance on examination tasks that overlapped with training activities. They do not indicate generalizable skill acquisition.

## 2 The case: a within-instructor counterbalanced pilot

### 2.1 Design and setting

This study employed a mixed-methods quasi-experimental design, combining quantitative outcome assessment with qualitative feedback collection, within a within-instructor counterbalanced framework. This feasibility pilot used a within-instructor counterbalanced design and was conducted at Jiangsu University School of Medicine from September to November 2024. The study was reported in accordance with the transparent reporting of evaluations with nonrandomized designs (TREND) statement (5).

The research procedure followed a sequential flow: (1) instructor recruitment and randomization of teaching model order; (2) student recruitment and informed consent; (3) baseline assessment; (4) implementation of Block 1 (Weeks 1–4) with four instructors teaching OBE and four teaching control; (5) immediate transition to Block 2 (Weeks 5–8) with model crossover for all instructors; (6) post-intervention comprehensive examination and self-assessment; (7) qualitative feedback collection; and (8) data analysis. No washout period was implemented between blocks.

The Research Ethics Committee of Jiangsu University Affiliated Hospital approved the study (KY2024K0106; approved 15 August 2024). All participants provided written informed consent. Non-participation would not affect academic grades, and withdrawal was permitted without penalty.

**Figure 1:**
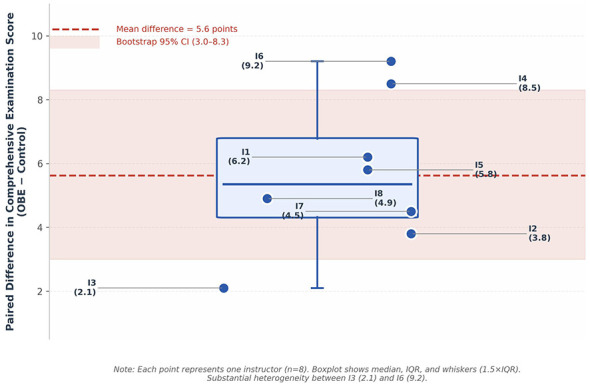
Instructor-level paired differences in comprehensive examination scores (OBE minus control). Each point represents one instructor (*n* = 8). Boxplot shows median, interquartile range, and whiskers (1.5 x IQR). Mean difference (5.6 points) indicated by dashed line. Bootstrap 95% CI (3.0–8.3) shown as shaded band. Individual instructor differences: I1 (6.2), I2 (3.8), I3 (2.1), I4 (8.5), I5 (5.8), I6 (9.2), I7 (4.5), I8 (4.9). No instructor showed negative differences. Note the substantial heterogeneity between I3 (2.1 points) and I6 (9.2 points). Even if statistical power were adequate, this variability might reflect random error or class effects rather than a true instructor-by-model interaction.

**Figure 2:**
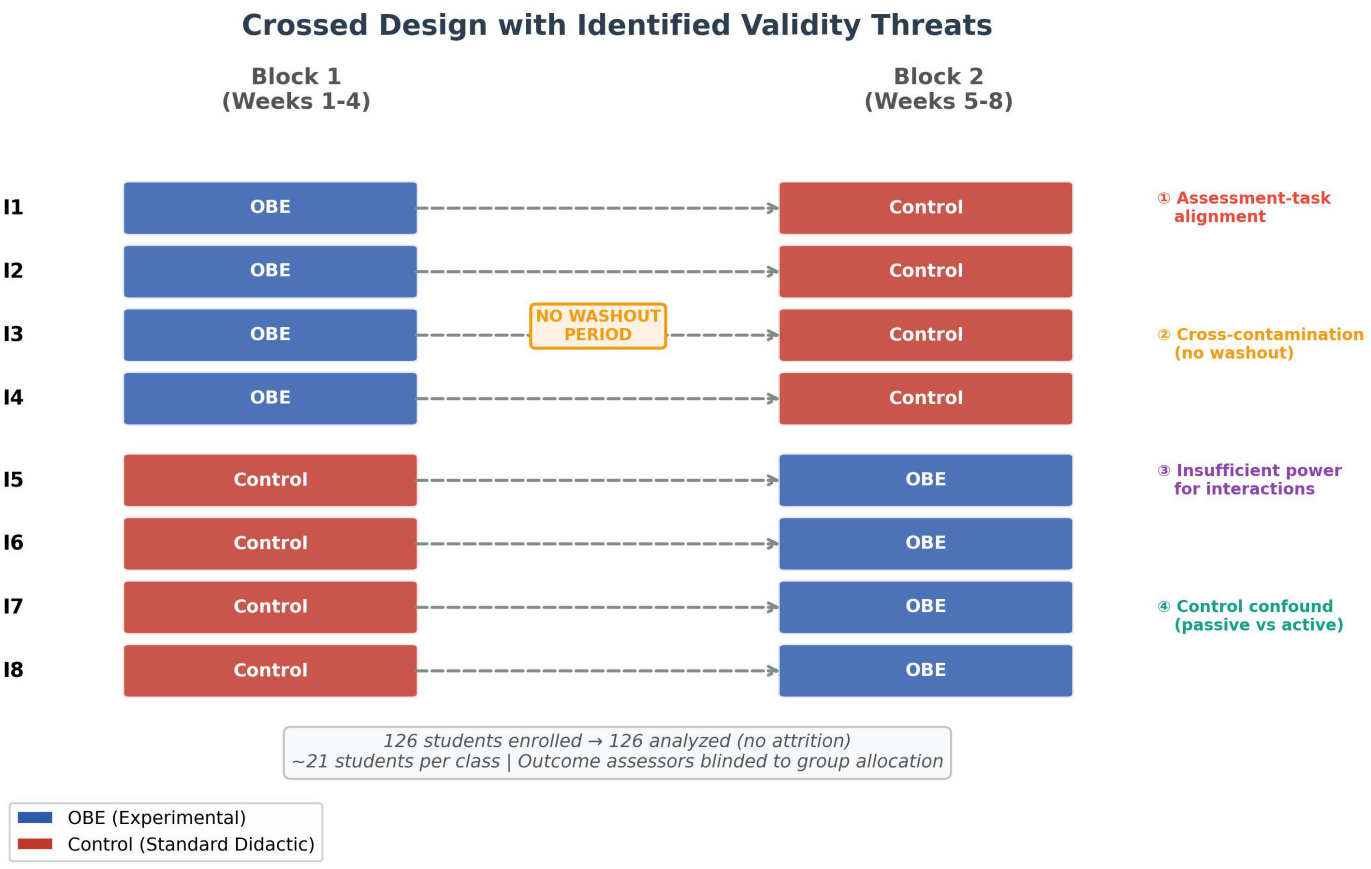
Schematic of the crossed design with identified validity threats. Eight instructors (I1–I8). Each instructor taught two classes over two consecutive four-week blocks. In block 1, I1–I4 taught OBE (experimental) while I5–I8 taught control; in block 2, I1–I4 taught control while I5–I8 taught OBE. Order of models was counterbalanced. No washout period was implemented between blocks. Validity threats identified: (1) assessment-task alignment between research design subscale and OBE micro-projects (PRIMARY THREAT); (2) potential cross-contamination due to absence of washout period; (3) insufficient power to detect instructor-by-model interactions (*n* = 8); (4) control condition confound (passive vs. active learning). Outcome assessments were conducted at end of each block. Student enrolment and analysis flow: 126 students enrolled, 126 analyzed (no attrition). Each class comprised approximately 21 students.

The study was registered at Research Registry (researchregistry7485) on 1 September 2024, before enrolment of the first participant. Research Registry is a prospective study registry but is not recognized as a primary registry by the World Health Organization International Clinical Trials Registry Platform (ICTRP). We acknowledge this limitation: the absence of WHO accreditation reduces transparency and traceability. For any study claiming causal or generalizable conclusions about educational interventions, registration at a WHO-recognized primary registry with a pre-uploaded statistical analysis plan is mandatory before enrolment of the first participant. Figures 1, 2 illustrate the instructor-level score differences and the crossed study design with identified validity threats, respectively.

### 2.2 Participants and instructors

All fifth-year clinical medical students rotating through the nephrology department were invited. Inclusion criteria were enrolment in the mandatory 8-week nephrology rotation and provision of informed consent. Exclusion criteria were previous participation in structured research training programs, defined as: (a) completion of a formal research methodology course with curricular credit; (b) participation in a structured undergraduate research program with faculty mentorship; or (c) prior authorship on a peer-reviewed publication. 126 students from six classes were enrolled, with no attrition.

Eight nephrology instructors participated (five associate professors and three lecturers; mean teaching experience 7.5 [SD 3.1] years; mean prior student ratings 4.1 [SD 0.4] out of 5.0; mean research engagement score 4.0 [SD 0.6] out of 5.0). Each instructor taught two classes over two consecutive 4-week blocks. Within each instructor, teaching model order was counterbalanced: four instructors began with OBE in block 1 and switched to standard in block 2, while the remaining four began with standard and switched to OBE. Assignment of starting model was determined by computer-generated random numbers (R v4.3). Because students were assigned by class according to the existing schedule rather than individually randomized, this remains a non-randomized study. No washout period was incorporated between blocks.

Students were unaware of the alternative teaching model. Outcome assessors were blinded to group allocation.

### 2.3 Intervention and control

The following describes the specific practices implemented in each group.

Experimental group (OBE model):The OBE research-integrated model was designed using backward-design principles (6), integrating four research elements over the 4-week block (eight sessions, 2 h each): (i) research-question-led instruction using unresolved clinical issues; (ii) platform-supported inquiry through virtual simulation and anonymized electronic health record databases; (iii) dynamic content updates on recent advances; and (iv) diverse research activities including structured micro-experiments, seminars, and clinical projects. Students completed a capstone mini-research proposal (6–8 h over 2 weeks, contributing 20% to final grade). The NephroSim virtual simulation platform presented 12 case scenarios covering common nephrology conditions, each with 8–12 decision nodes and immediate formative feedback. Intervention fidelity was ensured through standardized instructor training (a 4-h workshop), detailed manuals, and direct observation of 20% of sessions by an independent rater using a 12-item fidelity checklist. Mean fidelity score was 86% (range 78%−94%), with inter-rater agreement (Cohen kappa = 0.84).

Control group (standard didactic teaching): The control group received standard didactic teaching (eight lectures, 2 h each) focusing on established knowledge transmission through lectures without structured research activities, virtual platforms, or capstone projects. This condition comprised primarily passive lecture delivery, without the active learning components, virtual simulation, or research-integrated activities present in the experimental group.

The control condition was standard didactic teaching, which comprised primarily passive lecture delivery. This raises a fundamental confound: any observed differences may reflect active vs. passive learning rather than OBE *per se* (3, 7). We did not control for instructional time or cognitive engagement, and we acknowledge that this comparison conflates multiple pedagogical dimensions. Even if far-transfer assessments had been included, the active-vs.-passive-learning confound would remain unaddressed. Future studies must employ an active learning control condition to isolate the specific contribution of OBE's backward-design structure.

### 2.4 Outcome measures

The following assessment instruments were used, with their validity characteristics documented.

The primary outcome was the final comprehensive examination score (100-point scale) comprising knowledge recall (40%), case analysis (30%), and research design (30%). This examination was developed by four nephrology educators from other institutions, blinded to group allocation. The Content Validity Index (CVI) was 0.92, indicating excellent content validity. The examination demonstrated acceptable internal consistency (Cronbach's alpha = 0.84) and test-retest reliability (*r* = 0.78, *P* < 0.001) in a pilot sample of 30 students not included in the main study.

The research design subscale (30% of the examination) directly overlapped with OBE group micro-project activities. This overlap is structural and cannot be eliminated through statistical adjustment alone. Students in the OBE group practiced research design skills during micro-projects and were subsequently tested on similar tasks, whereas control students had no such practice. This creates a near-transfer assessment that conflates training exposure with competency acquisition (8). We conducted a sensitivity analysis excluding this subscale, but even the remaining subscales (knowledge recall, case analysis) may share implicit content with OBE instructional materials. No far-transfer assessment was included. Readers should interpret all effect estimates as upper bounds of near-transfer benefit, not evidence of generalizable competency improvement.

Secondary outcomes were: (i) pre- and post-intervention scores on the Medical Research Competency Self-Rating Scale (20 items, 5-point Likert, Cronbach alpha = 0.86); this instrument was adapted from Yoon et al. (9) and demonstrated content validity through expert panel review (CVI = 0.88) and construct validity through confirmatory factor analysis (CFI = 0.94, RMSEA = 0.06); (ii) blinded rating of capstone research proposals (OBE group only) by two independent researchers (ICC = 0.81); the rating rubric was developed based on the NIH grant review criteria and demonstrated inter-rater reliability (ICC = 0.81) and content validity (CVI = 0.90);(iii) learning satisfaction questionnaire (5-point Likert); (iv) System Usability Scale for the NephroSim platform; and (v) open-ended qualitative feedback.

### 2.5 Statistical analysis

Instructor-level paired analysis was the primary approach. For each instructor, we calculated the mean outcome for the OBE class and the control class, then applied a paired *t*-test to the eight instructor-level difference scores. The Wilcoxon signed-rank test was used as a non-parametric sensitivity analysis. Normality of difference scores was assessed with the Shapiro–Wilk test (*W* = 0.91, *P* = 0.37). Effect sizes are reported as Hedges g with 95% confidence intervals.

Non-parametric bootstrap (10,000 resamples, BCa) estimated 95% confidence intervals. Leave-one-out sensitivity analysis iteratively removed each instructor. Because the study was not designed to detect instructor-by-model interactions (power approximately 45% for a moderate interaction with 8 instructors) (10), we did not perform any interaction analysis.

For the five dimensions of research competency, no adjustment for multiple comparisons was applied because the analysis is exploratory. Missing data (2.4%) were handled by multiple imputation by chained equations (20 imputations) under the missing at random assumption. A complete-case analysis gave essentially identical results (data not shown).

With eight instructors, the design provides approximately 80% power for a paired Cohen *d* = 0.50 (alpha = 0.05). However, the small instructor sample precludes reliable estimation of between-instructor heterogeneity.

All analyses were conducted using R v4.3. The pre-specified statistical analysis plan (dated 1 September 2024) and complete analysis code are available through the Open Science Framework.

### 2.6 Results

Table 1 presents instructor characteristics. No significant differences existed between groups in baseline characteristics (all *P* > 0.05). Attendance rates were similar (OBE: 94.5%; control: 95.0%).

**Table 1:** Instructor characteristics (*N* = 8, anonymised by identifier).

Identifier	Rank	Subspecialty	Teaching experience (years)	Publications (last 5 years)	Prior student ratings (/5)	Research engagement score	Fidelity score (%)	Started with OBE
I1	Associate professor	Glomerular disease	11	7	4.5	4.8	91	Yes
I2	Lecturer	Dialysis	4	2	3.8	3.4	78	Yes
I3	Lecturer	Transplantation	5	2	3.9	3.6	82	Yes
I4	Associate professor	Tubulointerstitial	9	5	4.2	4.2	88	Yes
I5	Associate professor	Glomerular disease	10	6	4.3	4.5	94	No
I6	Lecturer	Dialysis	3	1	3.7	3.2	79	No
I7	Associate professor	Transplantation	8	4	4.1	4.0	86	No
I8	Associate professor	Tubulointerstitial	10	5	4.3	4.2	90	No
Mean (SD)	–	–	7.5 (3.1)	4.0 (2.3)	4.1 (0.4)	4.0 (0.6)	86.0 (5.8)	–

Table 2 presents primary outcomes from instructor-level paired analysis. The paired *t*-test showed that near-transfer assessment scores were higher in the OBE condition (mean difference 5.6 points, 95% CI 3.1–8.1, *P* = 0.001). The Wilcoxon signed-rank test confirmed this finding (*W* = 0, *P* = 0.008). Bootstrap (10,000 resamples) gave a mean difference of 5.6 points (95% BCa CI 3.0–8.3). Leave-one-out sensitivity analysis gave mean differences from 4.8 to 6.1 points, all *P* < 0.01.

**Table 2:** Primary outcomes and self-rated competency changes from instructor-level paired analysis.

Panel A: Near-transfer assessment scores^a^					
Outcome	OBE group (*n* = 63)	Control group (*n* = 63)	Adjusted β (95% CI)	*p*-value	Hedges' g (95% CI)
Comprehensive score	80.9 ± 8.0	75.3 ± 9.1	5.6 (3.1, 8.1)	0.001	0.67 (0.30, 1.04)
Knowledge recall	32.6 ± 4.3	31.0 ± 5.0	1.6 (0.9, 2.3)	0.001	0.35 (0.01, 0.69)
Case analysis	25.9 ± 4.7	20.0 ± 5.2	5.9 (3.3, 8.5)	<0.001	0.84 (0.46, 1.22)
Research design	22.4 ± 4.6	15.2 ± 4.3	7.2 (4.0, 10.4)	<0.001	1.01 (0.61, 1.41)
Panel B: Changes in self-rated research competency^b^					
**Dimension**	**OBE pre**	**OBE post**	**Control pre**	**Control post**	**Adjusted hedges' g (95% CI)**
Hypothesis formulation	2.8 ± 0.7	4.1 ± 0.6	2.9 ± 0.8	3.2 ± 0.7	0.81 (0.42, 1.20)
Evidence-based reasoning	2.9 ± 0.6	4.0 ± 0.7	2.8 ± 0.7	3.1 ± 0.6	0.73 (0.35, 1.11)
Literature search/appraisal	3.1 ± 0.8	3.9 ± 0.6	3.0 ± 0.7	3.3 ± 0.7	0.59 (0.22, 0.96)
Experimental design	2.6 ± 0.9	3.8 ± 0.8	2.7 ± 0.8	2.9 ± 0.8	0.62 (0.25, 0.99)
Statistical analysis	2.4 ± 0.8	3.6 ± 0.9	2.5 ± 0.7	2.8 ± 0.8	0.65 (0.28, 1.02)

^a^Mean difference from paired *t*-test on eight instructor-level difference scores. Sensitivity analysis (excluding research design subscale): mean difference 3.8 points (95% BCa CI 1.2–6.4, P = 0.005), representing a 32% attenuation. ^b^Mean (SD) shown. Hedges g from paired *t-*test on eight instructor-level difference scores for gain scores (post minus pre). OBE, outcome-based education; SD, standard deviation; CI, confidence interval.

These estimates are derived from near-transfer assessments that overlap with training content. They do not indicate generalizable research competency.

Sensitivity analysis excluding the research design subscale yielded a reduced but significant mean difference of 3.8 points (95% BCa CI 1.2–6.4, *P* = 0.005). This 32% attenuation suggests that a substantial portion of the observed effect may reflect assessment-task alignment rather than genuine competency transfer. This more conservative estimate (Hedges g approximately 0.45) may better reflect transferable benefit, though it remains subject to other biases.

Table 2 also shows pre- and post-test scores for research competency self-ratings. The OBE group showed larger gains across all five dimensions. The largest effect sizes were for hypothesis formulation (*g* = 0.81, 95% CI 0.42–1.20) and evidence-based reasoning (*g* = 0.73, 95% CI 0.35–1.11). Blinded assessment of capstone proposals (OBE group only) gave a mean score of 14.2 (SD 2.1) out of 20.

These self-rated competency gains and capstone scores reflect near-transfer performance. Without far-transfer assessment, they cannot be interpreted as evidence of generalizable skill acquisition.

Student satisfaction was high (mean 4.6 out of 5.0). The NephroSim platform yielded a mean System Usability Scale score of 72.4 (SD 11.6). Median usage was 3.2 (IQR 2.1–4.8) hours per student; 89% (56/63) completed all 12 scenarios at least once.

Correlation analysis showed modest positive associations between platform usage metrics and academic outcomes. Total scenario completion time correlated with comprehensive examination score (*r* = 0.31, 95% CI 0.08–0.51, *P* = 0.009). Hint request frequency showed a negative correlation with examination score (*r* = −0.24, 95% CI−0.45–−0.01, *P* = 0.04).

## 3 Why the design failed to deliver credible evidence

### 3.1 The fatal flaw: assessment-task alignment

The most serious threat to internal validity in this pilot was assessment-task alignment. The research design subscale of the comprehensive examination (30% of the total score) directly overlapped with the micro-project activities completed only by the OBE group. This is not a statistical nuisance that can be adjusted away; it is a structural feature of the study design.

Barnett and Ceci's taxonomy of transfer distinguishes between near transfer, where the training and assessment contexts are similar, and far transfer, where they differ substantially across content and context dimensions (8). In this pilot, the assessment was near-transfer by design: students practiced research design skills during micro-projects and were subsequently tested on essentially the same tasks. The observed differences cannot be distinguished from task-specific practice effects.

The sensitivity analysis excluding the research design subscale reduced the mean difference from 5.6 to 3.8 points, a 32% attenuation. Yet even this more conservative estimate remains difficult to interpret. The knowledge recall and case analysis subscales, while not directly overlapping with micro-projects, likely shared implicit content with OBE instructional materials. Without a far-transfer assessment, we cannot distinguish between task-specific practice effects and genuine competency acquisition.

### 3.2 Why counterbalancing did not rescue validity

The within-instructor counterbalanced design was intended to separate intervention effects from instructor-specific factors. In theory, having each instructor teach both models should partial out instructor main effects. In practice, this design failed for three reasons.

First, no washout period was implemented between blocks. Students within the same instructor may have discussed content across blocks, and instructors may have unconsciously transferred pedagogical techniques from the OBE model to the control condition in block 2. A *post-hoc* comparison of block 1 vs. block 2 scores showed no significant order effect (mean difference 1.2 points, 95% CI−2.1–4.5, *P* = 0.45), but this analysis is underpowered and should not be interpreted as evidence of no contamination. The risk of cross-contamination cannot be ruled out.

Second, with only eight instructors, the design was theoretically capable of separating instructor main effects but practically incapable of supporting causal inference. The power to detect a moderate instructor-by-model interaction is approximately 45%, far below acceptable thresholds (10). Consequently, the counterbalanced design added negligible value over a simple parallel design: we cannot determine whether the observed differences reflect a genuine model effect or an instructor-by-model interaction. Even if power were adequate, the substantial variability in individual instructor differences (range 2.1–9.2 points) might reflect random error or class effects rather than a true interaction.

Third, all eight instructors had above-average research engagement (mean 4.0 out of 5.0). Generalizability to instructors with limited research experience is uncertain. The sample was not representative of the broader instructor population, and the counterbalancing only addressed instructor main effects, not instructor-by-model interactions.

### 3.3 The control condition confound

Standard didactic teaching in this context comprised primarily passive lecture delivery without structured research activities. This raises the possibility that observed differences reflect active vs. passive learning rather than OBE *per se* (3, 7). We did not control for instructional time, cognitive engagement, or student autonomy. The comparison conflates multiple pedagogical dimensions, making it impossible to attribute any observed differences specifically to the OBE model. Crucially, the absence of far-transfer assessment means we cannot disentangle the specific benefits of OBE's backward-design and research-integration from the generic effects of active learning. Even if far-transfer assessments had been employed, the active-vs.-passive confound would still preclude any conclusion about OBE-specific effects.

### 3.4 Ethical justification for publishing a failed design

This study was initiated as a feasibility pilot before all design limitations were fully appreciated. During data analysis, the severity of assessment-task alignment and other validity threats became apparent. Rather than suppressing these findings, we chose to publish this methodological cautionary tale for three reasons. First, the medical education literature contains numerous studies with similar design flaws that nonetheless claim competency improvement; our transparency may prevent other teams from repeating these errors. Second, null or flawed studies are systematically underreported, creating publication bias that overestimates intervention effectiveness (11). Third, this article reports no effectiveness conclusions; it reports only methodological lessons. We believe this honest accounting serves the research community better than silent abandonment.

## 4 What would make future studies credible?

### 4.1 The non-negotiable requirement: graduated transfer assessment

The single most important improvement for future studies is the inclusion of graduated transfer assessment tasks. Drawing on Barnett and Ceci's taxonomy (8), we propose a three-tier framework that is more feasible for undergraduate medical education than immediate far-transfer tasks:

#### 4.1.1 Near transfer (the present study)

Assessment tasks identical in format and content to training activities. This level is necessary for formative feedback but insufficient for summative claims of generalizable competency.

#### 4.1.2 Intermediate transfer

Assessment tasks that retain the same underlying cognitive structure but apply it to novel clinical contexts. For example, students trained to design a nephrology study proposal could be asked to critique a study design from cardiology or gastroenterology, preserving the research design skill while changing the knowledge domain.

#### 4.1.3 Far transfer

Assessment tasks that differ from training across multiple dimensions simultaneously, such as reviewing an actual grant application or conducting a peer review of a manuscript from an unfamiliar specialty, administered 3–6 months post-rotation.

This graduated framework can be adapted to institutions with varying resource levels. For well-resourced institutions, all three tiers can be implemented simultaneously, with far-transfer assessment administered through partnerships with other departments or institutions. For resource-limited settings, intermediate transfer tasks can be prioritized as the minimum standard, using existing examination materials from other clinical rotations or collaborating with neighboring institutions to share assessment resources. The key principle is that assessment tasks must differ from training activities in at least one dimension (knowledge domain, physical context, temporal context, functional context, social context, or modality), regardless of institutional resources.

We recommend that future studies include at least intermediate transfer assessments as the primary outcome, with far transfer as a secondary outcome. This graduated approach balances scientific rigor with feasibility for fifth-year medical students. Table 3 provides a concrete template.

**Table 3:** Template for far-transfer assessment tasks in research competency evaluation.

Dimension of Barnett & Ceci taxonomy	Near-transfer (to be avoided)	Intermediate transfer (minimum standard for future studies)	Far-transfer (recommended for confirmatory studies)
Knowledge domain	Design a study proposal for a nephrology condition discussed during the rotation	Critically appraise a published study from cardiology or gastroenterology and identify methodological flaws	Review a grant application from an unfamiliar specialty and provide written peer review
Physical context	Complete assessment in the same classroom/lab setting	Complete assessment in a different department or simulation center	Complete assessment via remote platform
Temporal context	Assessment at end of rotation (immediate)	Assessment at end of rotation with altered item order	Assessment 3–6 months post-rotation
Functional context	Academic assessment for course credit	Authentic task: evaluate a mock grant proposal for methodological soundness	Team-based review of a real manuscript with students from different training levels
Social context	Individual completion	Pair completion with a student from a different rotation	Team-based completion with interdisciplinary peers
Modality	Written examination	Oral presentation or structured debate	Structured OSCE station with standardized patient

Intermediate transfer tasks should differ from training activities across at least one dimension; far-transfer tasks should differ across at least three dimensions. OSCE, objective structured clinical examination.

### 4.2 A methodological checklist for future studies

Based on the failures identified in this pilot, we propose the following checklist for future studies of research competency interventions:

(a) Graduated transfer assessment is mandatory. The primary outcome must include at least one assessment task at the intermediate transfer level, differing from training activities in knowledge domain while preserving the core skill structure. Far-transfer assessment is recommended as a secondary outcome.
(b) Instructor-by-model interactions must be estimable. The instructor sample must be sufficient to detect moderate instructor-by-model interactions with at least 80% power. Based on conventional power calculations for crossed random-effects models, this typically requires 12–16 instructors or more (10, 12).
(c) Washout periods or parallel designs must be used. If a counterbalanced design is employed, a minimum 2-week washout period between blocks is essential to reduce cross-contamination. Alternatively, a parallel-group design with randomization at the student or class level may be preferable, though this requires doubling instructor resources.
(d) Control conditions must isolate the intervention. The control condition should match the experimental condition on all dimensions except the specific intervention component under evaluation. An active learning control, such as case discussions plus literature review with equivalent time-on-task but without backward-design or research-integration components, is mandatory to disentangle OBE-specific effects from generic active learning benefits.
(e) Registration at a WHO-recognized primary registry is mandatory for confirmatory studies. Pre-uploaded protocols and statistical analysis plans enhance transparency. For any study claiming generalizability, registration at a WHO-recognized primary registry (such as the Chinese Clinical Trial Registry or ClinicalTrials.gov) before enrolment of the first participant is mandatory, with the statistical analysis plan uploaded at the time of registration.
(f) Time-on-task must be controlled or measured. If the intervention requires additional student time, this must be either controlled for in the design or measured and adjusted for in analysis.

### 4.3 Implications for the medical education research community

This case illustrates a broader problem in medical education research: the widespread use of near-transfer assessments to claim competency improvement. When assessment content structurally overlaps with intervention activities, statistically significant findings are essentially uninterpretable as evidence of generalizable skill acquisition (13). We urge the medical education research community to prioritize graduated transfer assessments in future studies of competency interventions.

Our findings align with and extend previous methodological critiques in healthcare professions education. Downing's seminal work on assessment validity emphasized that meaningful interpretation of assessment data requires evidence that the assessment captures the intended construct rather than task-specific familiarity (13). Similarly, van der Vleuten and Schuwirth's programmatic assessment framework argued that single-point assessments, especially those aligned with training content, cannot support competency claims (14). Recent work by Cook and Hatala on sample size adequacy in health professions education research highlighted that underpowered designs frequently produce spurious significant findings that cannot be replicated (10). Our study adds to this literature by demonstrating, through a concrete case, how assessment-task alignment specifically threatens validity even in designs that appear methodologically rigorous (within-instructor counterbalancing, blinded assessors, pre-specified analysis plans).

The resources required for credible evaluation are substantial. Far-transfer assessment development requires multidisciplinary expertise in education, psychometrics, and clinical content (14). Larger instructor samples require multi-center collaboration. Washout periods or parallel designs extend study timelines. These requirements may explain why many studies settle for near-transfer assessments, but the scientific cost is high: uninterpretable findings that cannot guide practice.

Based on the recommendations presented in this manuscript, we outline the key design features of a future multicenter confirmatory study: (1) Multi-center recruitment across 4–6 institutions to achieve a minimum of 16 instructors, providing adequate power to detect instructor-by-model interactions; (2) Randomization at the class level within each institution, with stratification by instructor; (3) An active learning control condition (case-based learning with equivalent time-on-task) to isolate OBE-specific effects from generic active learning benefits; (4) Graduated transfer assessment as the primary outcome, including intermediate transfer (critique of study designs from unfamiliar specialties) and far transfer (peer review of actual manuscripts, administered 3–6 months post-rotation); (5) A 2-week washout period between blocks if a counterbalanced design is used, or preferably a parallel-group design to eliminate cross-contamination; (6) Registration at a WHO-recognized primary registry (e.g., Chinese Clinical Trial Registry) with pre-uploaded statistical analysis plan before enrolment; (7) Pre-specified statistical analysis with crossed random-effects models to account for instructor-by-model interactions; and (8) Blinded outcome assessment with assessment tasks developed by educators from external institutions.

Recent evidence from China underscores the urgency of this issue. A national survey of 17,451 medical graduates found that research engagement did not necessarily enhance interest in integrating research into future careers, and only 27.1% of graduates had participated in research projects (15). Another study of 90 undergraduates in Guangdong identified lack of time (65.5%), insufficient guidance (56.6%), and limited resources (47.8%) as major barriers to research engagement (16). These findings highlight the gap between curricular research training and genuine competency development, reinforcing the need for rigorous evaluation designs that can distinguish between near-transfer performance and generalizable skill acquisition.

## 5 Conclusion

This methodological cautionary tale demonstrates that even carefully designed within-instructor counterbalanced studies can produce uninterpretable effect estimates when assessment content structurally overlaps with intervention activities. The pilot reported here showed higher near-transfer assessment scores in the OBE condition, but these differences cannot be interpreted as evidence of generalizable competency acquisition. The fatal flaws were assessment-task alignment, the absence of graduated transfer assessment, an insufficient instructor sample to detect interactions, and the lack of a washout period.

We provide no evidence that the OBE model improves research competency. We can only state that, in this pilot, students who completed micro-projects subsequently scored higher on examination tasks resembling those projects. This is a near-transfer effect of uncertain educational value.

The contribution of this article is to identify and warn against a common but dangerous validity threat, and to offer a concrete, actionable framework for avoiding it. Future studies must incorporate graduated transfer assessments, sufficient instructor samples to detect interactions, washout periods or parallel designs, and active control conditions that isolate the intervention. Multi-center replication with these features, plus mandatory registration at a WHO-recognized primary registry before enrolment, is required before any generalizable or causal conclusions can be drawn.

## Data Availability

The original contributions presented in the study are included in the article/supplementary material, further inquiries can be directed to the corresponding author.
