# Supplementary material for: When assessment alignment masks generalizability: a methodological cautionary tale from an OBE pilot in nephrology
Source: Front Med (Lausanne). 2026 Jul 28;13:1893135. doi: 10.3389/fmed.2026.1893135 (PMC13458752; doi:10.3389/fmed.2026.1893135)
Supplement: Supplementary file 1 [file Table_1.docx]

**SUPPLEMENTARY MATERIALS**

**When Assessment Alignment Masks Generalizability:
A Methodological Cautionary Tale from an OBE Pilot in Nephrology**

Qi-shun Wu¹,* · Lin Wang² · Gui-rui Sang¹

¹Second Affiliated Hospital of Wannan Medical University, Wuhu, Anhui, China
²Affiliated Fourth Hospital of Jiangsu University, Zhenjiang, Jiangsu, China

**Corresponding Author: Qi-shun Wu | E-mail: wqs1109@163.com*

**Table of Contents**

**Supplementary Material 1**: Fidelity Checklist for OBE Research-Integrated Teaching Model (12 items)

**Supplementary Material 2**: Methodological Checklist for Future Studies of Research Competency Interventions (6 items)

**Supplementary Material 3**: Statistical Analysis Plan Summary (8 components)

**Supplementary Material 4**: TREND Statement Checklist for Nonrandomized Evaluations (18 items)

**Supplementary Material 5**: R Analysis Code and Data Availability Statement

**Supplementary Material 6**: Informed Consent Form Template

**Supplementary Material 1**

**Fidelity Checklist for OBE Research-Integrated Teaching Model**

**Instructions:** Independent raters observed 20% of sessions and scored each item as Present (Yes) or Absent (No). Inter-rater agreement was assessed using Cohen kappa.

| **Item** | **Present (Yes/No)** | **Notes** |
| --- | --- | --- |
| 1. Instructor follows standardized lesson plan |  |  |
| 2. Research-question-led instruction implemented |  |  |
| 3. Virtual simulation platform (NephroSim) utilized |  |  |
| 4. Dynamic content updates on recent advances provided |  |  |
| 5. Structured micro-experiments conducted |  |  |
| 6. Seminars with student-led discussion held |  |  |
| 7. Clinical projects with formative feedback completed |  |  |
| 8. Capstone mini-research proposal guidance provided |  |  |
| 9. Backward-design principles evident in session structure |  |  |
| 10. Predefined competencies explicitly referenced |  |  |
| 11. Formative assessment integrated during sessions |  |  |
| 12. Student autonomy and inquiry encouraged |  |  |

***Note:*** *Mean fidelity score = 86% (range 78%–94%). Inter-rater agreement (Cohen kappa) = 0.84.*

**Supplementary Material 2**

**Methodological Checklist for Future Studies of Research Competency Interventions**

*This checklist is derived from the validity threats identified in the pilot study (Section 4.2 of the main text). All six items were absent in the present pilot and are proposed as mandatory or recommended requirements for future confirmatory studies.*

| **Checklist Item** | **Requirement Details** | **Present in Pilot** | **Priority** |
| --- | --- | --- | --- |
| **(a) Graduated transfer assessment is mandatory** | Primary outcome must include at least one assessment task at the intermediate transfer level (differing from training activities in knowledge domain while preserving core skill structure). Far-transfer assessment is recommended as a secondary outcome. | No | Critical |
| **(b) Instructor-by-model interactions must be estimable** | The instructor sample must be sufficient to detect moderate instructor-by-model interactions with at least 80% power. Based on conventional power calculations for crossed random-effects models, this typically requires 12–16 instructors or more. | No | Critical |
| **(c) Washout periods or parallel designs must be used** | If a counterbalanced design is employed, a minimum 2-week washout period between blocks is essential to reduce cross-contamination. Alternatively, a parallel-group design with randomization at the student or class level may be preferable. | No | High |
| **(d) Control conditions must isolate the intervention** | The control condition should match the experimental condition on all dimensions except the specific intervention component under evaluation. An active learning control (e.g., case discussions plus literature review with equivalent time-on-task but without backward-design or research-integration components) is mandatory. | No | Critical |
| **(e) Registration at WHO-recognized primary registry is mandatory** | For confirmatory studies, pre-uploaded protocols and statistical analysis plans enhance transparency. Registration at a WHO-recognized primary registry (e.g., Chinese Clinical Trial Registry, ClinicalTrials.gov) before enrolment of the first participant is mandatory. | No | High |
| **(f) Time-on-task must be controlled or measured** | If the intervention requires additional student time, this must be either controlled for in the design or measured and adjusted for in analysis. | No | Medium |

***Note:*** *Priority levels: Critical = essential for validity; High = strongly recommended; Medium = desirable if feasible.*

**Supplementary Material 3**

**Statistical Analysis Plan Summary**

*Pre-specified statistical analysis plan dated 1 September 2024.* All analyses were conducted using R version 4.3. The complete analysis code is available through the Open Science Framework.

| **Analysis Component** | **Method** | **Pre-specified** | **Deviation** |
| --- | --- | --- | --- |
| **Primary analysis** | Instructor-level paired t-test on eight instructor-level difference scores (OBE mean minus control mean per instructor). | Yes | None |
| **Non-parametric sensitivity** | Wilcoxon signed-rank test on the eight instructor-level difference scores. | Yes | None |
| **Bootstrap CI estimation** | Non-parametric bootstrap with 10,000 resamples using the bias-corrected and accelerated (BCa) method to estimate 95% confidence intervals. | Yes | None |
| **Leave-one-out sensitivity** | Iteratively remove each instructor and re-estimate the mean difference to assess influence of individual instructors. | Yes | None |
| **Interaction analysis** | Not performed. With only 8 instructors, power to detect a moderate instructor-by-model interaction is approximately 45%, far below acceptable thresholds. | Yes (excluded) | Power insufficient; analysis omitted per protocol |
| **Missing data handling** | Multiple imputation by chained equations (MICE) with 20 imputations under the missing-at-random (MAR) assumption. A complete-case sensitivity analysis was also performed. | Yes | None |
| **Multiple comparisons** | No adjustment for multiple comparisons applied because the analysis is exploratory and hypothesis-generating. | Yes | None |
| **Software** | R version 4.3. All code and the pre-specified SAP are available through the Open Science Framework. | Yes | None |

**Additional Specifications:**

**Power calculation:** With 8 instructors, the design provides approximately 80% power for a paired Cohen d = 0.50 (alpha = 0.05, two-tailed).

**Normality assessment:** Shapiro-Wilk test on instructor-level difference scores (W = 0.91, P = 0.37).

**Effect size:** Hedges g with 95% confidence intervals reported for all primary outcomes.

**Blinding:** Outcome assessors were blinded to group allocation. Examination developers were from other institutions and blinded to group allocation.

**Supplementary Material 4**

**TREND Statement Checklist for Nonrandomized Evaluations**

*This study was reported in accordance with the Transparent Reporting of Evaluations with Nonrandomized Designs (TREND) statement (Des Jarlais et al., Am J Public Health 2004;94:361-366). The following checklist documents compliance with each TREND item.*

| **Item** | **TREND Item Description** | **Location in Manuscript** | **Compliance** |
| --- | --- | --- | --- |
| **Title and abstract** |  |  |  |
| 1 | Indicate the study's nonrandomized design in the title | Title page | Yes |
| 2 | Provide a structured summary of study design, methods, results, and conclusions | Abstract | Yes |
| **Introduction** |  |  |  |
| 3 | State specific objectives, including any pre-specified hypotheses | **Introduction** | Yes |
| **Methods** |  |  |  |
| 4 | Describe the study design (e.g., within-instructor counterbalanced) | Section 2.1 | Yes |
| 5 | Describe the setting and locations | Section 2.1 | Yes |
| 6 | Describe eligibility criteria and sources of participants | Section 2.2 | Yes |
| 7 | Describe recruitment methods and period | Section 2.2 | Yes |
| 8 | Describe intervention and control conditions in sufficient detail | Section 2.3 | Yes |
| 9 | Describe outcomes and any diagnostic criteria | Section 2.4 | Yes |
| 10 | Describe sample size and power calculations | Section 2.5 | Yes |
| 11 | Describe blinding procedures | Section 2.2 | Yes |
| 12 | Describe statistical methods, including subgroup and interaction analyses | Section 2.5 | Yes |
| **Results** |  |  |  |
| 13 | Report participant flow using a diagram | Figure 2 (schematic) | Yes |
| 14 | Report dates defining periods of recruitment and follow-up | Section 2.1 | Yes |
| 15 | Report baseline characteristics | Table 1 | Yes |
| 16 | Report number analyzed for each outcome | Section 2.6 | Yes |
| 17 | Report outcome data and effect estimates with precision | Table 2 | Yes |
| **Discussion** |  |  |  |
| 18 | Interpret results considering study objectives, limitations, and generalizability | Sections 3–5 | Yes |

***Note:*** *TREND = Transparent Reporting of Evaluations with Nonrandomized Designs. All 18 items are addressed in the manuscript.*

**Supplementary Material 5**

**R Analysis Code and Data Availability Statement**

**1. Data Availability**

De-identified participant data, analysis code (R v4.3), and study materials are available from the corresponding author upon reasonable request. *The pre-specified statistical analysis plan (dated 1 September 2024) is available through the Open Science Framework (OSF).*

**2. Software and Packages**

**All analyses were conducted using** R version 4.3 (R Core Team, 2024). The following packages were used:

- **boot**: Bootstrap confidence interval estimation (10,000 resamples, BCa method)
- **mice**: Multiple imputation by chained equations (20 imputations)
- **lme4**: Crossed random-effects models (for power calculation reference)
- **ggplot2**: Figure generation
- **dplyr**: Data manipulation
- **readxl**: Data import

**3. Analysis Code Structure**

- **01_data_import.R**: Imports raw data, checks completeness, and performs initial data cleaning.
- **02_descriptive_stats.R**: Generates Table 1 (instructor characteristics) and baseline comparisons.
- **03_primary_analysis.R**: Performs instructor-level paired t-test and Wilcoxon signed-rank test.
- **04_bootstrap_ci.R**: Computes BCa bootstrap confidence intervals (10,000 resamples).
- **05_leave_one_out.R**: Iteratively removes each instructor and re-estimates mean differences.
- **06_self_rated_competency.R**: Analyzes pre-post changes in the five research competency dimensions (Table 2, Panel B).
- **07_sensitivity_analysis.R**: Excludes the research design subscale and re-estimates effects.
- **08_figures.R**: Generates all figures (Figures 1 and 2).

**4. Key Analysis Steps (Reproducible Summary)**

**Step 1.** Load de-identified dataset with instructor-level aggregated scores.

**Step 2.** Calculate paired differences: OBE class mean minus control class mean for each instructor (n = 8).

**Step 3.** Assess normality: Shapiro-Wilk test on paired differences (W = 0.91, P = 0.37).

**Step 4.** Primary analysis: Paired t-test on the 8 difference scores.

**Step 5.** Non-parametric sensitivity: Wilcoxon signed-rank test on the same 8 differences.

**Step 6.** Bootstrap: 10,000 resamples with BCa method for 95% CI.

**Step 7.** Leave-one-out: Iteratively remove I1–I8 and report range of mean differences.

**Step 8.** Self-rated competency: Calculate gain scores (post − pre) for each dimension, then paired t-test on instructor-level gain differences.

**Step 9.** Sensitivity analysis: Exclude research design subscale (30% of total score) and repeat primary analysis.

**5. Open Science Framework Repository**

**Project URL:** *[URL to be inserted upon OSF registration]*

**5. Open Science Framework Repository**

**Project URL:** *[URL to be inserted upon OSF registration]*

**Contents:** Pre-specified SAP (PDF), R analysis code (.R files), de-identified dataset (.csv), and this supplementary material.

**Supplementary Material 6**

**Informed Consent Form Template**

**Research Information**

**Study Title:** When Assessment Alignment Masks Generalizability: A Methodological Cautionary Tale from an OBE Pilot in Nephrology

**Principal Investigator:** Qi-shun Wu, MD, Second Affiliated Hospital of Wannan Medical University

**Co-Investigators:** Lin Wang, MD; Gui-rui Sang, MD

**Institution:** Jiangsu University School of Medicine

**Ethics Approval:** Research Ethics Committee of Jiangsu University Affiliated Hospital (KY2024K0106; approved 15 August 2024)

**Study Duration:** September to November 2024

**Registration:** Research Registry (researchregistry7485), registered 1 September 2024

**Purpose of the Study**

You are being invited to participate in a research study. This study is a feasibility pilot evaluating a new outcome-based education (OBE) research-integrated teaching model for nephrology rotations. *The primary purpose is methodological: to identify and warn against assessment-task alignment bias in educational intervention studies.* This study does not claim to demonstrate that the OBE model improves research competency.

**Study Procedures**

- Participation in either the OBE research-integrated teaching model or standard didactic teaching during your 4-week nephrology rotation block.
- Completion of a comprehensive examination (100 points) at the end of the rotation, comprising knowledge recall (40%), case analysis (30%), and research design (30%).
- Completion of the Medical Research Competency Self-Rating Scale (20 items, 5-point Likert) before and after the rotation.
- Completion of a learning satisfaction questionnaire and the System Usability Scale for the NephroSim platform (OBE group only).
- Optional submission of open-ended qualitative feedback.
- For the OBE group only: completion of a capstone mini-research proposal (6–8 hours over 2 weeks, contributing 20% to final grade).

**Risks and Benefits**

**Risks:** There are no anticipated physical risks. The primary risk is minimal inconvenience from completing questionnaires. All data will be de-identified.

**Benefits:** There is no direct personal benefit. Your participation will contribute to methodological knowledge about how to design valid educational intervention studies.

**Confidentiality**

All data will be de-identified. Your name will be replaced with a unique code number. Aggregated results will be reported; no individual will be identifiable in any publication. Data will be stored securely for 5 years and then destroyed.

**Voluntary Participation**

**Your participation is entirely voluntary. Non-participation will not** affect your academic grades or clinical rotation evaluation in any way. You may withdraw at any time without penalty. If you withdraw, your data will be excluded from analysis.

**Statement of Consent**

I have read and understood the above information. I have had the opportunity to ask questions, and all my questions have been answered to my satisfaction. I voluntarily agree to participate in this study.

| **Participant Name (print):** Lin Wang; Gui-rui Sang, | Date:2026-5-27 |
| --- | --- |
| **Signature:** | Date: 2026-5-27 |
| **Investigator Name (print):** Qi-shun Wu | Date: 2026-5-27 |

*— End of Consent Form —*
